# Draft Genome Sequence of Streptococcus agalactiae KALRO-LC1 Strain Isolated from a Mastitis-Infected Camel in Laikipia County, Kenya

**DOI:** 10.1128/mra.00910-22

**Published:** 2022-09-19

**Authors:** Edwin Murungi, Ednah Masila, Irene Ogali, Nathan Langat, Ruth Onywera, Vincent Malonza, Christine Inguyesi, Frank Onyambu, Hezron Wesonga, Monicah Maichomo

**Affiliations:** a Department of Medical Biochemistry, Kisii University, Kisii, Kenya; b Veterinary Research Institute, Kenya Agricultural and Livestock Research Organization, Muguga, Kenya; c School of Health Sciences, Meru University of Science and Technology, Meru, Kenya; University of Rochester School of Medicine and Dentistry

## Abstract

We report the draft genome sequence of Streptococcus agalactiae KALRO-LC1 strain obtained from a mastitis-infected camel in Laikipia County, Kenya. The 2,201,604-bp draft genome is assembled into 3 contigs with a GC content of 35.87% and is predicted to contain 1,192 protein-coding sequences.

## ANNOUNCEMENT

Streptococcus agalactiae (Group B Streptococcus) causes mastitis in camels resulting in diminished milk production and consequently enormous economic losses that imperil the livelihoods of pastoralists in northern Kenya (1). Moreover, S. agalactiae causes serious infections in humans (2, 3). The pathogen is therefore a threat to human and veterinary health.

We report the draft genome of a S. agalactiae KALRO-LC1 isolate recovered from a mastitis-infected camel in Laikipia County, Kenya. Milk samples were inoculated on blood agar containing 5% sheep blood and incubated overnight at 37°C. Colonies with zones of beta-hemolysis, and that were catalase negative and Gram positive, were cultured in Edward’s selective media to ascertain genus type (4). Lancefield grouping confirmed the isolate as Streptococcus agalactiae (5). The isolated bacteria were cultured on nutrient agar plates by incubation under aerobic conditions at 37°C for 16 h followed by enrichment of fresh single colonies in 100 mL nutrient broth and incubation under aerobic conditions at 37°C for 16 h with rotation at 200 rpm (6) to a cell density of 0.5 to 0.7 at OD_600_ (7). Genomic DNA was extracted using the QIAamp DNA kit (Qiagen) according to manufacturer’s instructions. An Oxford Nanopore Technologies (ONT) sequencing library was then prepared using the manufacturer’s Native Barcoding Expansion 1-12 (EXP-NBD104) in conjunction with the Ligation Sequencing kit (SQK-LSK109), and sequencing was performed on a MinION device using R9.4.1 flow cell to give 33,183 raw reads of *N*_50_ 103 kb. Basecalling of the raw sequence data (fast5) and subsequent demultiplexing was performed using GUPPY v3.6.1 (https://nanoporetech.com). Adaptors were trimmed using PORECHOP v0.2.4 (8), and NANOFILT v2.5.0 (9) was used for quality filtering by removing reads <500 bp or with average quality scores of <10. The draft genome was assembled using UNICYCLER v0.5.0 (10) and quality assessed using QUAST v5.2.0 (11). The contigs were annotated using NCBI's PGAP v6.1 (12). Default parameters were used for all software unless otherwise specified.

The assembly contained 3 contigs totaling 2,201,604 bp with a GC content of 35.87%, an *N*_50_ of 2,139,486 bp, and a coverage of 95×. Annotation revealed a total of 2,269 genes (1,192 coding genes), 23 rRNAs, 85 tRNAs, 3 ncRNAs, and 2 CRISPR arrays.

Using VaxiJen (13), IgPred (14), and AllerTOP v.2 (15), we have delineated several potentially viable antigenic vaccine candidates (Table 1). Our results will foster further investigations into the development of a subunit vaccine for S. agalactiae infections.

**TABLE 1:** Putative S. agalactiae antigenic vaccine candidates

NCBI sequence accession	VaxiJen score	Subcellular localization	Transmembrane helices	IgPred prediction	AllerTOP prediction
MCP9189840.1	1.0828	Cell wall	1	IgG Epitope	Probable nonallergen
MCP9189846.1	0.9236	Cytoplasmic membrane	1	IgG Epitope	Probable nonallergen
MCP9189915.1	0.7253	Unknown	0	IgG Epitope	Probable nonallergen
MCP9189959.1	0.7667	Cytoplasmic	0	IgG Epitope	Probable nonallergen
MCP9189976.1	0.7542	Unknown	0	IgG Epitope	Probable nonallergen
MCP9189841.1	1.5040	Cytoplasmic	0	IgG Epitope	Probable nonallergen
MCP9189875.1	1.1086	Unknown	0	IgG Epitope	Probable nonallergen
MCP9190210.1	1.0150	Unknown	0	IgG Epitope	Probable nonallergen
MCP9190214.1	1.0590	Unknown	1	IgG Epitope	Probable nonallergen
MCP9190551.1	1.0850	Cytoplasmic membrane	0	IgG Epitope	Probable nonallergen
MCP9190938.1	1.0042	Cytoplasmic membrane	1	IgG Epitope	Probable nonallergen
MCP9190954.1	1.0033	Unknown	0	IgG Epitope	Probable nonallergen
MCP9190991.1	1.1004	Extracellular	0	IgG Epitope	Probable nonallergen
MCP9189842	0.9959	Cell wall	0	IgG Epitope	Probable nonallergen
MCP9190131.1	0.9931	Unknown	0	IgG Epitope	Probable nonallergen
MCP9190265.1	0.9462	Cytoplasmic	0	IgG Epitope	Probable nonallergen
MCP9190286.1	0.9441	Cytoplasmic membrane	4	IgG Epitope	Probable nonallergen
MCP9190290.1	0.9461	Cytoplasmic membrane	1	IgG Epitope	Probable nonallergen

### Data availability.

This Whole Genome Shotgun project has been deposited at DDBJ/ENA/GenBank under accession JANCLS000000000. Raw sequence reads are available at the Sequence Read Archive (SRA) database under accession SRX16447265.
